# Dual-pulse photoactivated atomic force microscopy

**DOI:** 10.1038/s41598-021-96646-4

**Published:** 2021-08-24

**Authors:** Byullee Park, Seunghyun Lee, Jimin Kwon, Woojo Kim, Sungjune Jung, Chulhong Kim

**Affiliations:** Departments of Electrical Engineering, Medical Device Innovation Center, Convergence IT Engineering, Mechanical Engineering, Pohang University of Science and Technology (POSTECH), Pohang, 37673 Republic of Korea

**Keywords:** Nanoscale devices, Imaging and sensing

## Abstract

Photoactivated atomic force microscopy (pAFM), which integrates light excitation and mechanical detection of the deflections of a cantilever tip, has become a widely used tool for probing nanoscale structures. Raising the illuminating laser power is an obvious way to boost the signal-to-noise ratio of pAFM, but strong laser power can damage both the sample and cantilever tip. Here, we demonstrate a dual-pulse pAFM (DP-pAFM) that avoids this problem by using two laser pulses with a time delay. The first laser heats the light absorber and alters the local Grüneisen parameter value, and the second laser boosts the mechanical vibration within the thermal relaxation time. Using this technique, we successfully mapped the optical structures of small-molecule semiconductor films. Of particular interest, DP-pAFM clearly visualized nanoscale cracks in organic semiconductor films, which create crucial problems for small-molecule semiconductors. DP-pAFM opens a promising new optical avenue for studying complex nanoscale phenomena in various research fields.

## Introduction

In recent years, many research areas, particularly the life and materials sciences, have increasingly used nano-analytical techniques that can observe nanoscale or atomic structures^1–4^. For example, super-resolution florescence microscopy, stimulated emission depletion microscopy^5^, and stochastic optical reconstruction microscopy^6^, can all observe details of cell structures that previously could not be resolved by conventional light microscopy. Electron microscopy^7^, another nano-analytical instrument that uses electrons of far shorter wavelengths than light, can characterize nanostructured semiconductors and polymers. Atomic force microscopy (AFM), one of the most powerful tools for characterizing and imaging material surfaces at the nanoscale, offers resolution on the order of a few nanometers, far exceeding the optical diffraction limit^8^. AFM-based optical tools are widely used for analyzing the surface, physical, and electrical properties of nanomaterials^9^. The optically induced AFM toolset includes infrared spectroscopy (AFM-IR)^10,11^, photo-induced force microscopy (PiFM)^12^, photothermal induced resonance (PTIR)^13^, photothermal atomic force microscopy (PT-AFM)^14^, and photoactivated atomic force microscopy (pAFM)^15^. These AFM techniques all detect the movement of a cantilever tip resulting from laser irradiation of the target, and various methods are being researched to improve their resolution and contrast. Conventional AFM-IR directly measures the cantilever tip’s vibration amplitude in response to a change in the wavelength of the light source. Its sensitivity is improved by using a laser with the same frequency as the contact resonance frequency of the cantilever tip. PiFM achieves excellent sensitivity when the frequency of the laser repetition rate is adjusted to match the difference in the first and second mechanical resonance frequencies of the cantilever tip. This matching also reduces the interaction between the tip and the sample when using non-contact AFM. PT-AFM measures the thermal expansion of a sample at nanoscale resolution in a similar way to the previously described techniques. In recently published study^16^ by Takahashi et al., they implemented variable frequency measurements by applying a multi-pulse modulation technique to observe microcrystalline Cu(In,Ga)(S,Se)2 [CIGSSe] materials. Super-resolution visible pAFM obtains images and captures the optical properties of materials smaller than 10 nm by using a cantilever tip and an optical excitation system. The first generation pAFM system improved its resolution by detecting the 2nd harmonics (at twice the repetition rate of the pulse laser) using a lock-in amplifier. All of the above AFM techniques can acquire and analyze optical spectroscopic data of such targets as polymer nanocomposites, cancer cells, and collagen, and can successfully obtain photoreactive images of nanoparticles and biological samples by using a specific light wavelength. However, despite their variety, the techniques all have limited SNR and contrast because the magnitude of the output signal is linearly related to the laser fluence, and increasing the laser power to improve the contrast can damage the sample and cantilever tip. Thus, the laser power is typically set to a preset threshold level.

In a parallel research field, a pump-probe beam method using two pulse lasers has been successfully introduced for photoacoustic (PA) imaging^17–26^ to improve the spatial resolutions and signal-to-noise ratio (SNR) of the received PA signal^27,28^. This method delivers two identical or two different short laser pulses to the targeted object, with a few microseconds delay between the pulses. The first laser pulse generates a signal from the target and increases the local temperature, and the second laser illuminates the thermally targeted region. Because the Grüneisen parameter is temperature-dependent, the second laser pulse produces a nonlinearly enhanced PA signal from the sample. This method has been adopted for improving the contrast and the axial and lateral resolutions of PA microscopy (PAM) imaging, and it has enabled PAM to obtain highly sensitive label-free nucleus and red blood cell images.

In this study, we apply pump-probe technology to existing pAFM to realize a new dual-pulse pAFM (DP-pAFM), that provides morphological information and highly sensitive optical characteristics of samples at several-nanometer resolution. Our newly developed DP-pAFM offers improved image contrast and sensitivity without exceeding the maximum power of previous single-pulse pAFM (SP-pAFM). Hence the risk of damaging or even destroying the target sample or the cantilever tip is minimized. Using DP-pAFM, we successfully mapped the optical properties of organic semiconducting materials with improved SNR and contrast. Particularly, organic or small-molecule semiconductors have attracted great attention for use in device displays and lighting applications, including organic light emitting diodes, thin film batteries, electronic paper, and wearable sensors because of their easy fabrication, low cost, mechanical flexibility, and applicability to plastic substrates^29^. One of the key problem when fabricating small-molecule semiconductors is the development of cracks during the film formation or annealing processes. The surface shape and any cracks formed during thermal expansion and bending affect the performance of low molecular weight semiconductors. In addition, cracks in organic semiconductor films are highly undesirable because they deteriorate charge transport. Previous studies have used conventional optical microscopes to observe these cracks^29–32^, but they are difficult to visualize beyond the optical diffraction limit. As semiconductor processes achieve increasingly fine line widths, the need to observe and understand nanoscale cracks becomes more and more pressing. The improved sensitivity and contrast of our proposed DP-pAFM system allowed us to identify the result of nanoscale cracks in a semiconducting film. Especially in small-molecule semiconductor films, fine cracks can significantly impact conductivity characteristics of the film-based organic semiconductor. The proposed DP-pAFM system could potentially extend the use of pAFM to research in a variety of new fields, such as surface component analysis, biological sample imaging, and ultrafine structure imaging at nanoscale.

## Methods

### Preparation of DPP-DTT and printing of soluble organic semiconductor materials

A donor–acceptor (D-A) polymer semiconductor ink, diketopyrrolo-pyrrole-dithiophene-thienothiophene (DPP-DTT), was prepared in a 1 mg·ml-1 solution using orthodichlorobenzene as a solvent (Sigma-Aldrich, St. Louis, United States). The DPP-DTT ink was filtered with a 0.45 μm PTFE filter and then printed inside a bank-guided rectangular area on a glass substrate using an air pulse dispenser (IMAGE MASTER 350PC, MUSASHI Engineering Inc., Tokyo, Japan) at 1 kPa dispensing pressure and 40° C plate temperature. After the ink dried, the sample was annealed for 30 min at 100° C. A small-molecule organic semiconductor ink, FlexOS (NeuDrive, Macclesfield, United Kingdom), was diluted in 10% solution using tetralin (Sigma-Aldrich, St. Louis, United States). The FlexOS ink was filtered with a 0.45 μm PTFE filter and then the ink was printed inside a bank-guided rectangular area on a glass substrate^33^, using an air pulse dispenser at 1 kPa dispensing pressure and a 40° C platen temperature. After the ink dried, the sample was annealed for 30 min at 100° C.

### Dual-pulse photoactivated atomic force microscopy system

**Figure 1:**
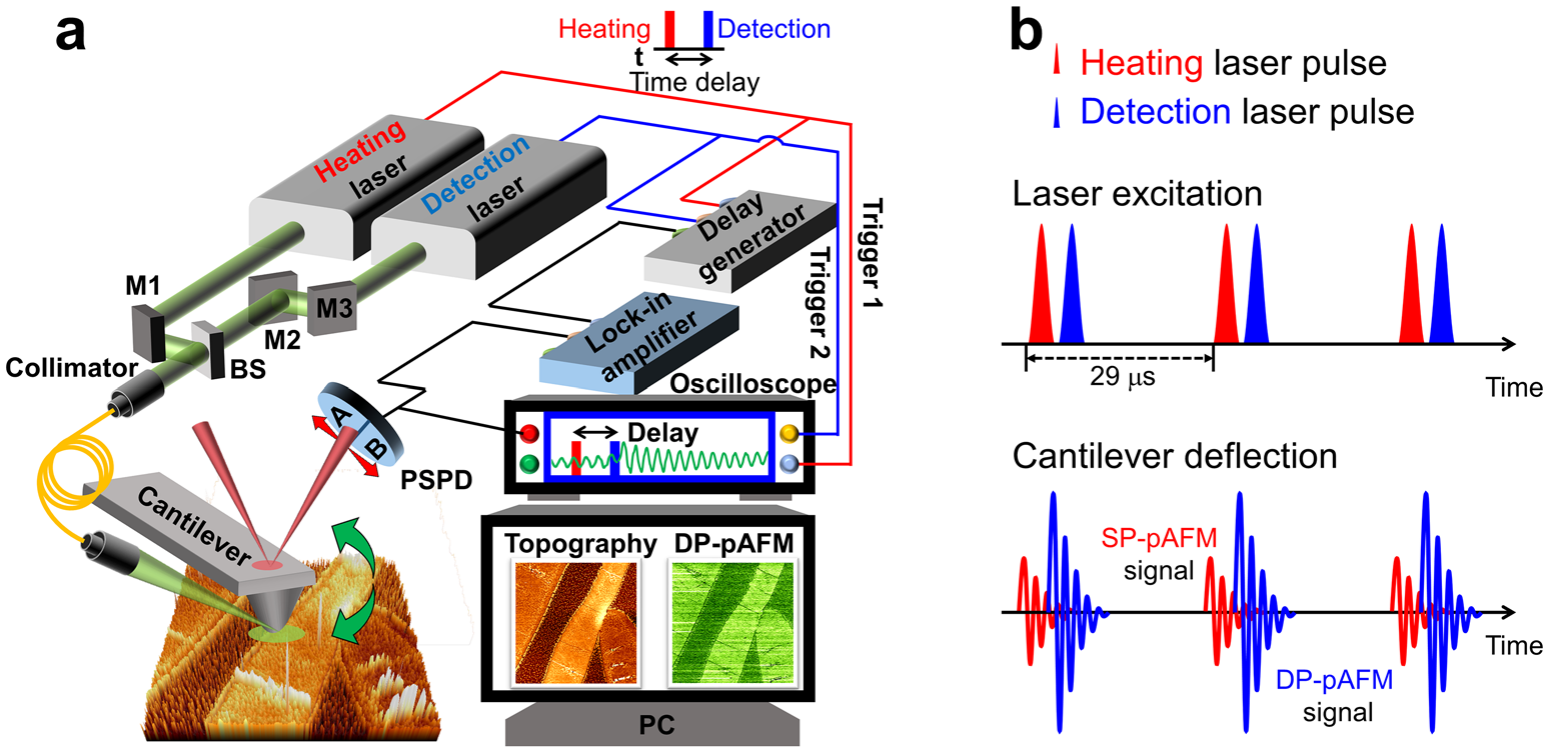
Dual-pulse photoactivated atomic force microscopy (DP-pAFM). (**a**) System schematic. (**b**) Generation of DP-pAFM signal. SP-pAFM, single-pulse; M, mirror; BS, beam splitter; PSPD, position-sensitive photodiode.

The DP-pAFM system, shown schematically in Fig. 1a, consisted of a Park Systems XE7 SP-pAFM (Suwon-si, Republic of Korea) and two Q-switched diode-pumped solid-state laser systems (SPOT-10-200-532, Elforlight, Daventry, UK)). One laser was used for heating the target and the other for detecting the target. The cantilever tip was an aluminum reflex coated tip (BudgetSensors, ContAl-G, Sofia, Bulgaria) with a tip radius of 10 nm and a contact force of 6 nN. The scanning speed was set to 0.2 Hz. Both lasers operated at 532 nm, with a pulse duration of 1.6 ns, and the pulse repetition rate was set to 34 kHz by an external trigger. The spot size of the beam irradiated on the sample was 30 μm. The existing SP-pAFM used only one laser source for photothermal expansion of the sample. The beam, transmitted through a single mode fiber, obliquely irradiated the sample. When irradiated by the laser pulses, the sample alternately absorbed a portion of the light and photothermally expanded, then contracted when the pulse was switched off, generating vibrations which deflected the cantilever. The cantilever vibration amplitude was proportional to the optical absorption coefficient of the sample and the laser pulse energy. For DP-pAFM, two laser beams were generated from the two identical lasers, separated by the time delay controlled by a digital delay generator (DG645, Stanford Research Systems, Sunnyvale, CA, USA). Using mirrors and a beam splitter, the combined beam was coupled into a single-mode fiber and illuminated obliquely onto the target sample through a focusing system composed of multiple optical lenses. Once the heating laser illuminated the target under the cantilever tip, the second laser illuminated the same area after a short delay. The resulting enhanced volume expansion of the sample caused cantilever oscillations unique to the characteristics of the sample. The cantilever vibration signals were captured by the position sensitive photodiode (PSPD), which detected changes in the beam reflected from the cantilever. The cantilever deflection signal from the PSPD was fed into the lock-in amplifier (SR830, Stanford Research Systems, USA). A pulse repetition rate of lasers was synchronized with the contact resonance frequency of the cantilever deflection via a delay generator, which was the reference signal of the lock-in amplifier. This laser setup is known as a "resonance-enhancing technique", which enhances the vibration of the cantilever, and efficiently detects light absorption, enabling more sensitive measurements^11,34,35^. The second harmonic response of the contact resonance as the lock-in output (pAFM signal) was used to minimize the background signal and increase the signal sensitivity^15^. The output signals of the lock-in amplifier were used as an input to an AFM controller for obtaining the topography and amplitude images.

## Results

### Principle of dual-pulse photoactivated atomic force microscopy (DP-pAFM)

In pAFM, once a target absorbs a pulse of light, its volume expands due to the thermoelastic and photo-thermal effects. The repeated rapid volume changes are expressed as ${\text{S}}\left( \lambda \right)$^36–38^:

1 $${\text{S}}\left( {\uplambda } \right) = {{ \Gamma \upeta }}_{{{\text{th}}}} {\upmu }_{{\text{a}}} \left( {\uplambda } \right){\text{F}},$$

where ${\Gamma }$ is the Grüneisen value, heat conversion efficiency ${\upeta }_{{{\text{th}}}}$, ${\upmu }_{{\text{a}}} \left( {\uplambda } \right)$ is the optical absorption coefficient of a sample at an wavelength $\left( {\uplambda } \right)$, and optical fluence ${\text{F}}$. The value of the Grüneisen parameter of the sample is a dimensionless element proportional to the mechanical stress converted from thermal energy and is denoted by

2 $${\Gamma } = \frac{{{{\upalpha \upnu }}_{{\text{s}}}^{2} }}{{{\text{C}}_{{\text{P}}} }},$$

where thermal expansion coefficient α, speed of sound in the medium ${\upnu }_{{\text{s}}}$, and heat capacity of the sample ${\text{C}}_{{\text{P}}}$ at the pressure. Importantly, in nature $,{\upalpha }$, ${\upnu }_{{\text{s}}}$, and ${\text{C}}_{{\text{P}}}$ are very closely related to temperature, so ${\Gamma }$ has a very temperature-dependent characteristic.

In contact mode AFM, the volume change quickly causes the target to hit the cantilever tip, and the cantilever begins to vibrate at the contact resonance frequency of the tip. The path of the reflected beam from the top of the cantilever is also altered in the four-quadrant photodiode where the beam hits the target and produces the basic pAFM signal. As mentioned, the DP-pAFM system (Fig. 1a) uses two lasers: the heating laser provides the first pulse, and the detection laser provides the second pulse after a specific time delay. When the target under the cantilever tip is first irraditated by the heating laser at 532 nm, Eq. (1) can be re-written as

3 $${\text{S}}_{1} \left( {{\uplambda }_{1} } \right) = {{ \Gamma }}_{1} {\upeta }_{{{\text{th}}}} {\upmu }_{{\text{a}}} \left( {{\uplambda }_{1} } \right){\text{F}}_{1} ,$$

where the initial Grüneisen value is ${\Gamma }_{1}$, the optical absorption coefficient at the wavelength of the heating laser is ${\upmu }_{{\text{a}}} \left( {{\uplambda }_{1} } \right)$, and heating laser fluence is ${\text{F}}_{1}$. After the heating laser irradiation, the temperature-dependant Grüneisen parameter is changed and denoted by

4 $${\Gamma }_{2} \left( {{\text{T}}_{{\Delta t}} ;\Delta t} \right) = {{ \Gamma }}_{1} \left[ {1 + {\upbeta } \cdot\Delta {\text{T}} \cdot {\uptau }\left( {\Delta {\text{t}}} \right)} \right],$$

where ${\text{T}}_{{\Delta t}}$ is the local temperature of the target sample at time $\Delta t$ after irradiation with the heating laser, ${\upbeta }$ is a coefficient that relates the absorbed thermal energy in the first pulse to the Grueneisen parameter change upon the second laser excitation^27,39^, $\Delta {\text{T}}$ is the induced temperature rise, and ${\uptau }\left( {\Delta {\text{t}}} \right)$ is the thermal relaxation function in $\Delta t$. Note that $\Delta {\text{T}}$ after the heating laser irradiation can be approximated by

5 $$\Delta {\text{T }} = { }\frac{{{\upmu }_{{\text{a}}} \left( {{\uplambda }_{1} } \right){\text{F}}_{1} }}{{\rho C_{v} }},$$

where $\rho$ is the density, and $C_{v}$ is the heat capacity of the volume. After the time delay, the detection laser irradiates the sample, causing a second photo-thermal expansion, ${\text{S}}_{2} \left( {{\uplambda }_{2} } \right)$, which can be derived from the following expression (6–7):

6 $${\text{S}}_{2} \left( {{\uplambda }_{2} } \right) = {\Gamma }_{2} {\upeta }_{{{\text{th}}}} {\upmu }_{{\text{a}}} \left( {{\uplambda }_{2} } \right){\text{F}}_{2} = {{ \Gamma }}_{1} \left[ {1 + {\upbeta }\Delta {\text{T}} \cdot {\uptau }\left( {\Delta {\text{t}}} \right)} \right] \cdot {\upeta }_{{{\text{th}}}} \cdot {\upmu }_{{\text{a}}} \left( {{\uplambda }_{2} } \right){\text{F}}_{2} .$$

Equation (6) is organized into two terms as follows:

7 $${\text{S}}_{2} \left( {{\uplambda }_{2} } \right) = { }\left[ {{\Gamma }_{1} {\upeta }_{{{\text{th}}}} {\upmu }_{{\text{a}}} \left( {{\uplambda }_{2} } \right){\text{F}}_{2} } \right]_{{\text{A}}} + \left[ {{{\upbeta \Gamma }}_{1} {\upeta }_{{{\text{th}}}} \cdot \frac{{{\upmu }_{{\text{a}}} \left( {{\uplambda }_{1} } \right){\upmu }_{{\text{a}}} \left( {{\uplambda }_{2} } \right)}}{{\rho C_{v} }} \cdot {\uptau }\left( {\Delta {\text{t}}} \right) \cdot {\text{F}}_{1} {\text{F}}_{2} } \right]_{{\text{B}}} ,$$

where ${\uplambda }_{1}$ is equal to ${\uplambda }_{2}$, A is the base pAFM signal induced by the detection laser, and B is the nonlinearly increased pAFM signal from the alternation of laser fluences ${\text{F}}_{1}$ and ${\text{F}}_{2}$. The increased volume expansion creates stronger cantilever deflection and oscillation than when a heating laser is used only (Fig. 1b). The DP-pAFM signal is directly related to the optical absorption property of the sample and the optical fluence of the source, which means that samples with low absorption (i.e., the background) cannot produce highly amplified signals. In other words, compared to the background signal, a much-amplified signal can be obtained from a target with high optical absorption, so the contrast of the resulting image can be improved. With this improvement, DP-pAFM can provide valuable nanoscale images and information that SP-pAFM cannot.

### Comparative increases in SP-pAFM and DP-pAFM signal amplitudes with increasing heating laser power

**Figure 2:**
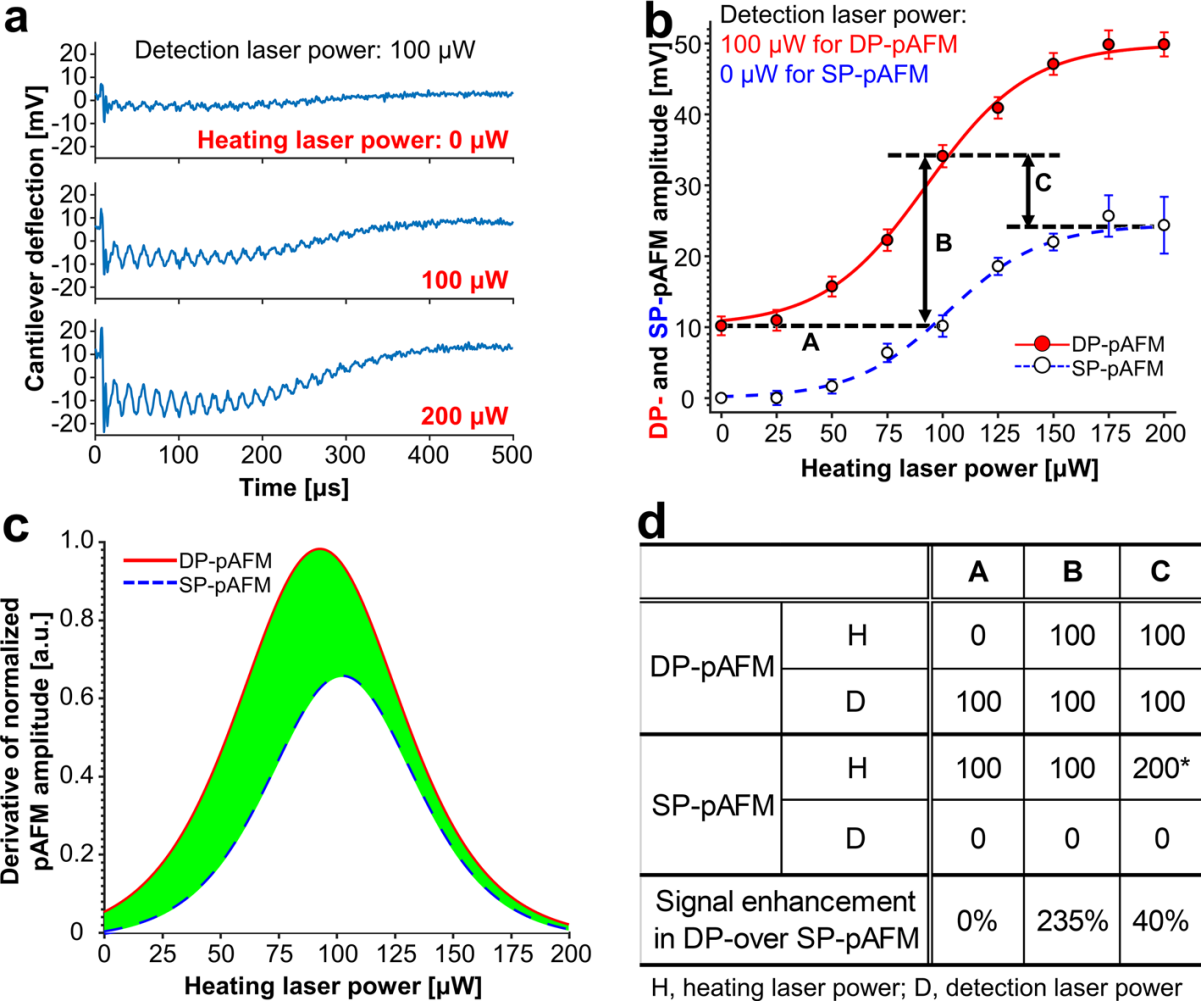
Increase in DP-pAFM and SP-pAFM signal amplitudes by changing the heating laser power. The detection laser powers for the DP-pAFM and SP-pAFM are fixed at 100 and 0 µW, respectively (**a**) Cantilever deflection of the position-sensitive photodiode (PSPD) at heating laser powers of 0, 100, and 200 µW. (**b**) Averaged DP-pAFM and SP-pAFM signal amplitudes with increasing heating laser power. (**c**) Derivatives of the normalized DP- and SP-pAFM amplitudes from (**b**). (**d**) Enhancement in DP-pAFM signal amplitude over SP-pAFM for three cases: A, B, and C. SP-pAFM, single-pulse pAFM; DP-pAFM, dual-pulse pAFM. *The sample and cantilever tip were damaged.

As shown in Fig. 1, the DP-pAFM system uses two identical 532-nm pulse lasers to generate alternating heating and detection pulses, separated by a variable time delay ranging from nanoseconds to microseconds. We measured both SP- and DP-pAFM signal amplitudes at increasing heating laser powers, and confirmed that, compared to the SP-pAFM values, the DP-pAFM signal amplitudes are significantly greater due to increased Grüneisen parameter value caused by the pre-heating pulse. For both systems, the time delay between the heating pulse and detection pulse was set to 10 ns. Likewise, the heating laser intensities for both systems were varied from 0 to 200 μW (i.e., 0–100 nJ/pulse). However, the detection laser intensity of the SP-pAFM was fixed at 0 μW, and that of the DP-pAFM was 100 μW (50 nJ/pulse). Since there was a sufficient time delay between the heating laser pulse and the detection laser pulse, it was reasonable to regard the laser energy inputs to the sample as two individual amounts rather than as their sum. For this experiment, the target was a highly absorbing polymer film of the semiconducting material diketopyrrolo-pyrrole-dithienylthieno [3,2-b]thiophene (DPP-DTT)^40^. As a baseline, we mesured the gradual increase in cantilever vibration detected by the PSPD with an increase in heating laser power by using the oscilloscope (Fig. 2a). The fast vibration is driven by the thermal expansion of the sample acting as a force impulse on the cantilever^11^. On the other hand, the slow vibrations may be caused by the system configuration where light is irradiated from an oblique direction above the sample, which may affect not only the sample but also the tip and cantilever. However, this slow vibration could be excluded through lock-in detection. Next we acquired SP- and DP-pAFM images of a DPP-DTT polymer sample with 160 × 32 data points (Fig. 1). We averaged all the signal amplitudes in each image to compare the difference between them (Fig. 2b) (blue circle and dashed line for SP-pAFM; red circle and solid line for DP-pAFM). As the heating laser power increases, the SP-pAFM amplitude also increases, reaches a peak at a heating laser power of 175 μW, and then falls a bit. Similarly, the DP-pAFM amplitude increases with increasing heating laser power and reaches a peak at 200 μW. Note that there is a 10 mV offset in the DP-pAFM amplitude owing to the preset detection laser power of 100 μW. The increase rates of the SP- and DP-pAFM amplitudes are compared with respect to the changes in heating laser power in Fig. 2c. As the heating laser power increases, both the SP- and DP-pAFM amplitudes likewise increase and peak at a heating laser power of around 100 μW. It is particularly noteworthy that the DP-pAFM signal amplitude rises more steeply than the SP-pAFM signal, and the derivative of the DP-pAFM amplitude at the peak point is about 46% higher than that of the SP-pAFM. After the peaks, both SP- and DP-pAFM signal amplitudes start becoming saturated at similar rates. The pAFM signal saturation might show nonlinear responses^41^. The signal enhancement in DP- over SP-pAFM (i.e., (DP_amp_ − SP_amp_)/SP_amp_ × 100) is summarized in Fig. 2d. In case A, the first column of the table, both DP- and SP-pAFM use the same laser power of 100 μW, so the enhancement in DP-pAFM amplitude is 0%. In case B, the DP-pAFM signal amplitude is enhanced by 235% over the SP-pAFM signal. This result proves that the dual laser pulses quite significantly enhance the DP-pAFM signal amplitudes. In case C, where the total laser powers used by both DP-pAFM and SP-pAFM are 200 μW, the DP-pAFM signal is 40% higher than the SP-pAFM signal. Interestingly, in repeated experiments, we found that an SP-pAFM heating laser power over 200 μW most certainly damaged the cantilever tip and DPP-DTT polymer surface, but DP-pAFM with heating and detection laser powers of 100 and 100 μW, respectively, did not. This finding confirmed our hypothesis that DP-pAFM can improve the SNR without damaging the sample or tip. Based on these results, the heating and detection laser intensities of the DP- and SP-pAFM were fixed at 100/100 μW and 100/0 μW, respectively in all subsequent experiments. The damage threshold power of the heating laser should be varied depending on the samples and cantilever tips.

### Changes in DP-pAFM signals versus time delays between heating and detection laser pulses

**Figure 3:**
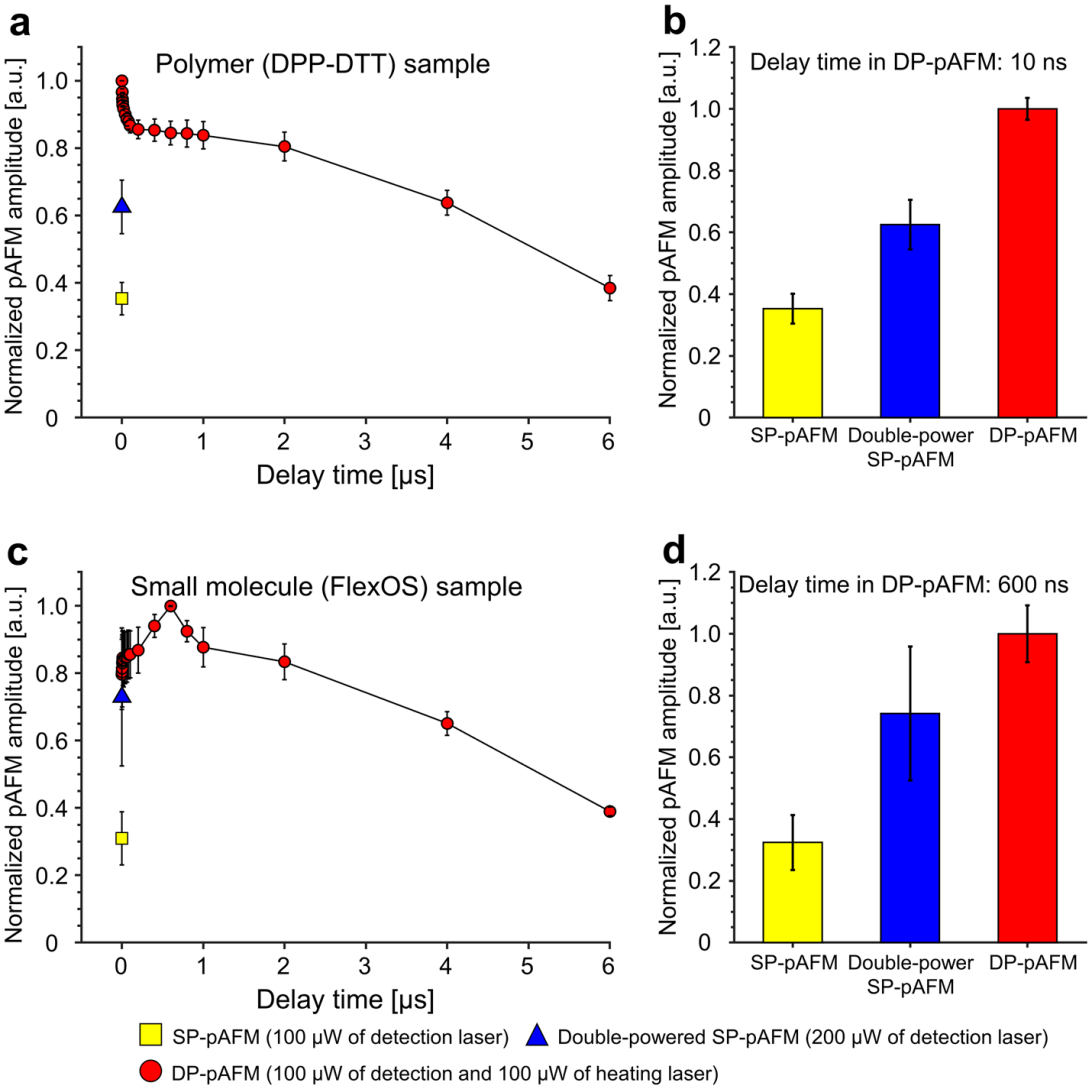
Comparison of the signal amplitudes for SP-pAFM, double-powered SP-pAFM, and DP-pAFM on two targets. (**a**,**b**) DPP-DTT polymer (n = 3) quantified signal amplitudes. (**c**,**d**) FlexOS small-molecule semiconductor film (n = 3) quantified signal amplitudes. SP-pAFM, single-pulse pAFM; DP-pAFM, and dual-pulse pAFM.

The degree of signal enhancement depends upon the time delay and is significant when the time delay is within the thermal relaxation time given by (8):

8 $${\uptau }_{{{\text{relax}}}} = { }\frac{{{\text{z}}^{2} }}{{{\text{k}}_{{{\text{eff}}}} }},$$

where ${\text{z}}^{2}$ is the heat travel distance and ${\text{k}}_{{{\text{eff}}}}$ is the thermal diffusivity of the sample, respectively^42,43^. In this study, we considered only the lateral direction of the thermal relaxation time because the thicknesses of the DPP-DTT polymer itself and of the small-molecule polymer film are much smaller than the laser spot size. We conducted experiments to find the optimal time delays between the heating and detection laser pulses for DPP-DTT and commercially available small-molecule semiconductor FlexOS films, with the results shown in Fig. 3 (n = 3 for each sample). To obtain the analysis result, each experiment was repeated 3 times for three different samples and the average value was used. The error bars denote the standard error obtained from the multiple experiments. To the best of our knowledge, no exact values for the thermal properties of DPP-DTT and FlexOS samples are available, so the thermal relaxation values were empirically derived from multiple experiments. Figure 3 shows the comparative signal amplitudes in DPP-DTT and FlexOS of SP-pAFM (i.e., 100 μW detection laser only), double-powered SP-pAFM (200 μW detection laser only), and DP-pAFM (100 μW detection laser and 100 μW heating laser). In the DPP-DTT sample, highly enhanced signals were observed at a delay of 0–200 ns (Fig. 3a, and Fig. S2 for enlarged graph). The DP- pAFM shows maximum amplitude when the delay is zero. Interestingly, the DP-pAFM signal is 60% higher than that of double-powered SP-pAFM at a delay of 0 ns, where they could be expected to be the same. We believe that the jittering, which always exists between pulses, might result in a short time delay, thus enhancing the DP-pAFM signal over the double-powered SP-pAFM signal. We conducted an experiment to confirm the jittering of the two laser pulses. Figure S3a shows a schematic for this experiment. We set the delay generator’s two trigger output delays to zero. After irradiating pulses from two lasers to each photodiode sensor, the photodiode signal was verified through an oscilloscope (Fig. S3b). As a result of box plotting the peak signals of photodiode 1 and 2 by acquiring the data measured with the oscilloscope a total of 10 times, we confirmed that there is jittering (Fig. S3c). The maximum time difference between peak values of each photodiode was 5.3 ns and 5.35 ns, respectively. The jittering of the two laser pulses is recorded in the Supplementary movie 1. In addition, the pulse width fluctuations were estimated based on this data, and as a result, the pulse width fluctuations of the two lasers were 1.6 ns and 1.5 ns, respectively. Then, the fluctuation of laser pulse energy was measured using an optical power meter and sensor. As a result, the pulse energy fluctuations of the two lasers were 1.1% and 0.24%, respectively. Next, a delay of 10 ns was selected to avoid the delivered laser energy being strengthened by any overlap of the two pulses (Fig. 3b). At 10 ns delay, the DP-pAFM signals are increased by 60% and 183% compared to the double-powered SP-pAFM and SP-pAFM, respectively. The DP-pAFM signal becomes similar to the double-powered SP-pAFM at around a 4 μs delay, and to SP-pAFM signals at around a 6 μs time delay. As shown in Fig. 3c, the temperature rise time of the FlexOS sample appears to be relatively slower than that of the DPP-DTT sample. This can be understood as a structural characteristic between samples, which may be because, unlike the crystal structure of FlexOS, the thermally activated charge of DPP-DTT with an amorphous structure was disturbed during intermolecular interaction, leading to a rapid temperature rise^44^. DP-pAFM with an optimal delay of 600 ns shows 34.8% and 208% signal increases compared to double-powered SP-pAFM and SP-pAFM, respectively (Fig. 3d). In both DPP-DTT and FlexOS samples, the DP-pAFM amplitude tends to decrease gradually, which could be influenced by the deflection of the cantilever by the heating laser pulse. The DP-pAFM signal becomes less strong than that of double-powered SP-pAFM after a delay of 3 μs. Of particular note, the double-powered SP-pAFM amplitudes acquired from the FlexOS sample are fluctuate widely, causing a large error bar. We confirmed that the samples were consistently impaired at a heating laser power of 200 μW. Overall, at the optimal time delay measured for all samples, the enhanced DP-pAFM signal is at least 2.8 times that of the SP-pAFM and at least 1.3 times that of the double-powered SP-pAFM.

We also compared the SP- and DP-pAFM signals of glass and DPP-DTT samples (Fig. S4). SP- and DP-pAFM images of a DPP-DTT sample on a glass substrate (Fig. S4a) were quantified using histograms of the signal amplitude (Fig. S4b). The averaged SP-pAFM signal from the DPP-DTT area is 299 ± 23 mV, and the averaged DP-pAFM signal is 947 ± 43 mV, which corresponds to a signal amplitude increase rate of 317% (Fig. S4c). The signal increase from the glass sample is comparatively lower, at only 273%. We calculated the image contrast and SNR of the DPP-DTT polymer sample. The contrasts for SP- and DP-pAFM were calculated as $\left( {{\text{S}}_{{{\text{DPP}} - {\text{DTT}}}} - {\text{ S}}_{{{\text{Glass}}}} } \right)/{\text{S}}_{{{\text{Glass}}}}$, where ${\text{S}}_{{{\text{DPP}} - {\text{DTT}}}}$ and ${\text{S}}_{{{\text{Glass}}}}$ represent the average signals in the DPP-DTT and glass regions, respectively, as seen in Fig. S4(a). The calculated image contrasts for SP- and DP-pAFM are 1.04 and 1.37, respectively. Thus, the image contrast of DP-pAFM is 32% better than that of SP-pAFM. In addition, the SNRs for SP- and DP-pAFM were calculated as ${\text{S}}_{{{\text{DPP}} - {\text{DTT}}}}$*/*${\upsigma }_{{{\text{DPP}} - {\text{DTT}}}}$, where ${\upsigma }_{{{\text{DPP}} - {\text{DTT}}}}$ represents the standard deviation in the DPP-DTT region in Fig. S4. The calculated SNRs for SP- and DP-pAFM are 24.3 dB and 32.0 dB, respectively. Thus, the SNR of DP-pAFM is 7.7 dB better than that of SP-pAFM. Using DP-pAFM, we observed that greater signal amplification appears in samples with optical properties of higher magnitudes, which increases the contrast of the DP-pAFM images. Compared to SP-pAFM, this enhanced DP-pAFM signal amplitude boosts the image contrast. In this result, the pAFM signal is confirmed even in glass with little light absorption, which shows that the tip itself is also affected when the pAFM laser irradiated the sample. However, the important point is that the increase in pAFM signal due to thermal expansion of the sample with high light absorption is more pronounced.

### Detection of cracks in a small-molecule semiconductor film using DP-pAFM

**Figure 4:**
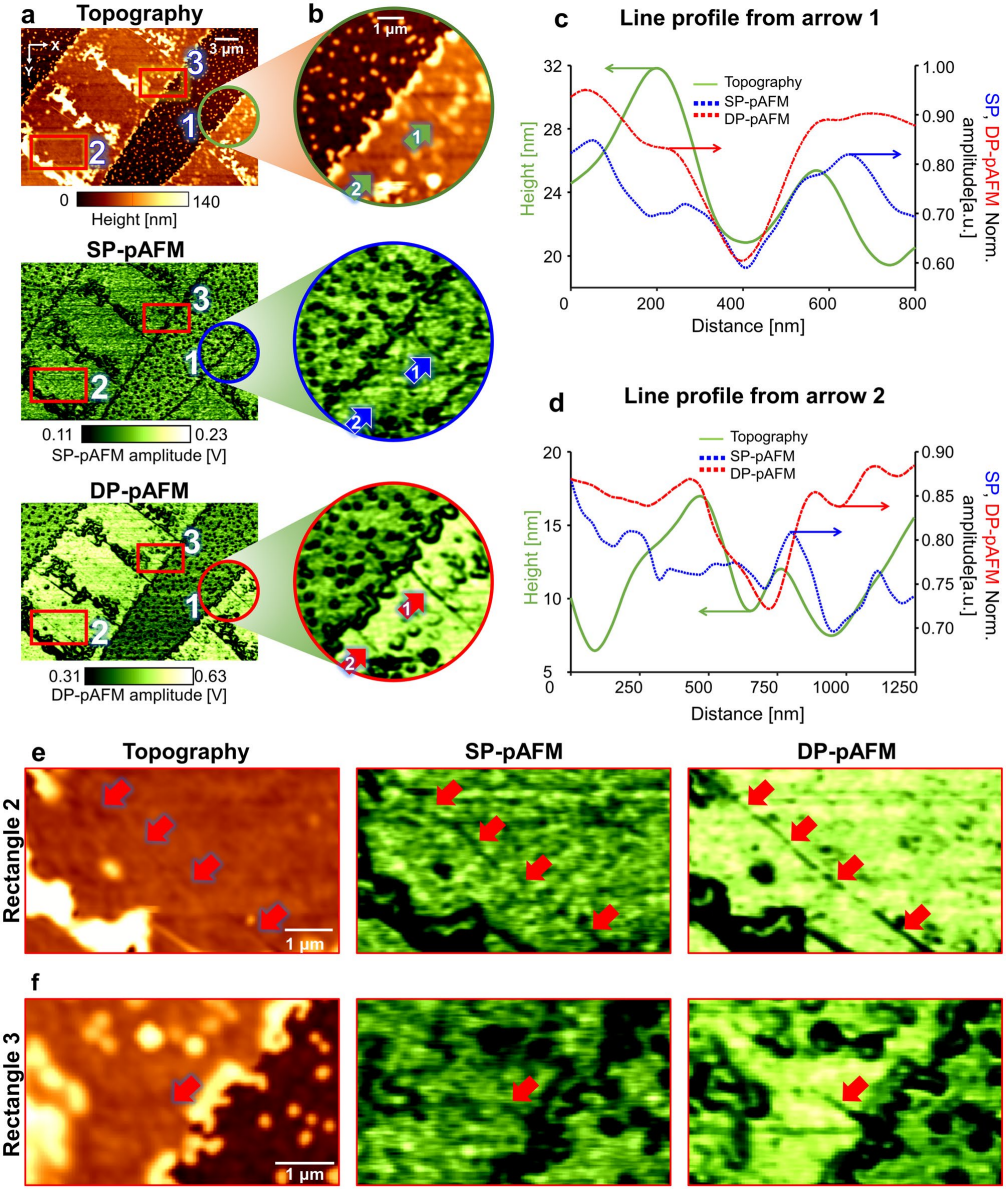
Analysis of nanosized cracks in a small-molecule organic semiconductor film. (**a**) AFM topographic and SP- and DP-pAFM amplitude images of the film. (**b**) Zoomed-in images of circle #1 in each image in (**a**). (**c**) Line 1-1′ profiles and (**d**) line 2-2′ profiles of panel (**b**). Zoomed-in images of (**e**) Rectangle #2 and (**f**) rectangle #3 in panel (**a**). Red arrows indicate a very fine crack. AFM, atomic force microscopy; SP-pAFM, single-pulse pAFM; DP-pAFM, dual-pulse pAFM; Norm, normalized.

To demonstrate the application of our proposed DP-pAFM system to an important industrial problem, we successfully observed nanoscale cracks in the FlexOS film that were difficult to see in AFM topographic images. Unlike other regions of the film, where small molecules with a strong optical absorption are distributed, a crack lacks small molecules and thus generates comparatively low amplitude pAFM signals. Over a 30 μm × 20 μm FOV, we obtained AFM topographic images and SP-pAFM and DP-pAFM signal amplitude images of the film. As seen in the topographic image in Fig. 4a, the crystallization of small molecules outside the crack region increased the target height by up to 30 nm over the non-crystallized region. In detail, the topographic image was divided into two areas: an crystallized region (i.e., the relatively bright part, height > 70 nm) and an non-crystallized region (i.e., the relatively dark part with spots, height ≅ 0 nm). The crystallized region was formed to a height of 70 nm or more, and the high-density crystallized region of the FlexOS material was formed to a height of 120 nm or more. On the other hand, many bright spots that are the result of nucleation of FlexOS are scattered in the non-crystallization region^45^. In the SP- and DP-pAFM amplitude images (Fig. 4a, SP-pAFM and DP-pAFM images), strong signal amplitudes were obtained in the crystallized regions and are expressed brightly. In particular, the DP-pAFM image has a wide signal range from bright green to dark, while the SP-pAFM image shows a relatively narrow signal range from dark green to dark, proving the enhanced image contrast of DP-pAFM. For better visualization, Fig. 4b shows cropped and zoomed-in images of Circle #1 in the topographic image and the SP-pAFM and DP-pAFM amplitude images in Fig. 4a. Along the 1–1′ line profile of Fig. 4b, the AFM image indicates a small-molecule crack that are lower than the surrounding region (Fig. 4c, green line). The crack is also imaged by SP-pAFM and DP-pAFM (Fig. 4c, blue dotted line and red dashed-dot line, respectively), but DP-pAFM provides a much clearer image with higher contrast than SP-pAFM. More interestingly, in the 2–2′ line profile trace of Fig. 4d, a suspected very fine crack that was barely detected with AFM and SP-pAFM (Fig. 4d, green line and blue dotted line, respectively) is clearly marked by high-contrast DP-pAFM (Fig. 4d, red dashed-dot line). Looking at the magnified topographic image (Fig. 4e) of Rectangle #2 (Fig. 4a), a very fine crack is faintly visible, as indicated by the red arrows. This crack is more clearly identifiable in the enlarged SP-pAFM image, but is noticeably discontinuous (SP-pAFM in Fig. 4e). A much more discernible image of crack is shown in the enlarged DP-pAFM image (DP-pAFM in Fig. 4e). This sequence of images shows that DP-pAFM can delineate a crack with superior contrast to conventional AFM topography and SP-pAFM imaging. As one more example, the crack in Square #3 (Fig. 4a) is detectable only in the enlarged DP-pAFM image, but not in the enlarged topographic and SP-pAFM images (Fig. 4f). Overall, it can be concluded that the DP-pAFM can image cracks much more clearly than conventional AFM or SP-pAFM, suggesting that DP- pAFM can be potentially used as a crack identifier during semiconductor fabrication processes. As mentioned before, cracks are morphological features that are caused during the film formation or annealing processes, and it common for AFM topography to show the cracks. However, if the width of the crack is narrower than the AFM resolution, cracks will not be detected by the conventional AFM. DP-pAFM, however, can detect very fine cracks because it can induce strong thermal expansion in those areas where small molecules are dense, and it also makes relative differences larger than those where small molecules are lacking.

## Discussion and conclusion

We present a new dual-pulse pAFM system that provides improved SNR and contrast without requiring laser energy beyond the damage threshold, thus preserving the sensitive cantilever tip as well as the light-absorbing target. DP-pAFM uses a pre-heating pulse to increase the local value of the Grüneisen coefficient, then, after a delay, sends a second pulse whose absorbtion boosts the mechanical vibration of the target for AFM detection. In DP-pAFM signal amplitude analysis experiments with increasing heating laser power and delay time change, DP-pAFM obtained much stronger signal amplitudes than SP-pAFM and double-powered SP-pAFM for all targets. We compared the SP-pAFM signal with the DP-pAFM signal within the thermal relaxation time. In addition, we demonstrated that the DP-pAFM could successfully image nanoscale cracks in a small-molecule semiconductor film with much better image contrast and SNR than SP-pAFM and AFM. We showed that DP-pAFM can achieve sharper images than SP-pAFM, with superior image contrast and SNR. Several criteria have been used to identify cracks in organic semiconductor films, but this study has the limitation that it did not prove that they are real cracks. This crack identification problem can be addressed by comprehensive consideration of the chemical, physical and electrical properties of the sample and can be addressed in future studies. These results demonstrate that DP-pAFM not only detects cracks that are difficult to identify with AFM, but also effectively solves the low image contrast and SNR problem of SP-pAFM. The enhanced DP-pAFM system is expected to be used for research in a variety of fields, from pure science to applied engineering.

## Supplementary Information

Supplementary Figures.

Supplementary Video 1.

Supplementary Legend.

## Data Availability

The datasets generated during and/or analysed during the current study are available from the corresponding author on reasonable request.
