# Supplementary material for: Dual-pulse photoactivated atomic force microscopy
Source: Sci Rep. 2021 Aug 24;11:17097. doi: 10.1038/s41598-021-96646-4 (PMC8384876; doi:10.1038/s41598-021-96646-4)
Supplement: Supplementary file 1 — Supplementary Figures. [file 41598_2021_96646_MOESM1_ESM.pdf]

Supplementary Information

Dual-pulse Photoactivated Atomic Force Microscopy

Authors

Byullee Park[1†], Seunghyun Lee[1†], Jimin Kwon[1], Woojo Kim[1], Sungjune Jung[1*], and Chulhong Kim[1*]

Affiliations

[1]Departments of Electrical Engineering, Convergence IT Engineering, Mechanical Engineering, and Medical Device Innovation Center, Pohang University of Science and Technology (POSTECH), Pohang, Republic of Korea 37673.

[†] These authors contributed equally to this work.

*Corresponding authors: chulhong@postech.edu and sjjung@postech.ac.kr

Figures S1-S4

- **Supplementary Figure 1:** DP- and SP-pAFM images of a DPP-DTT polymer sample, with 160 x 32 data points
- **Supplementary Figure 2:** Comparison of single-pulse (SP)-, double-powered single-pulse, and dual-pulse pAFM (DP-pAFM) signal amplitudes
- **Supplementary Figure 3:** Jittering measurement of heating and detection lasers
- **Supplementary Figure 4:** Comparison of SP and DP-pAFM signal amplitudes

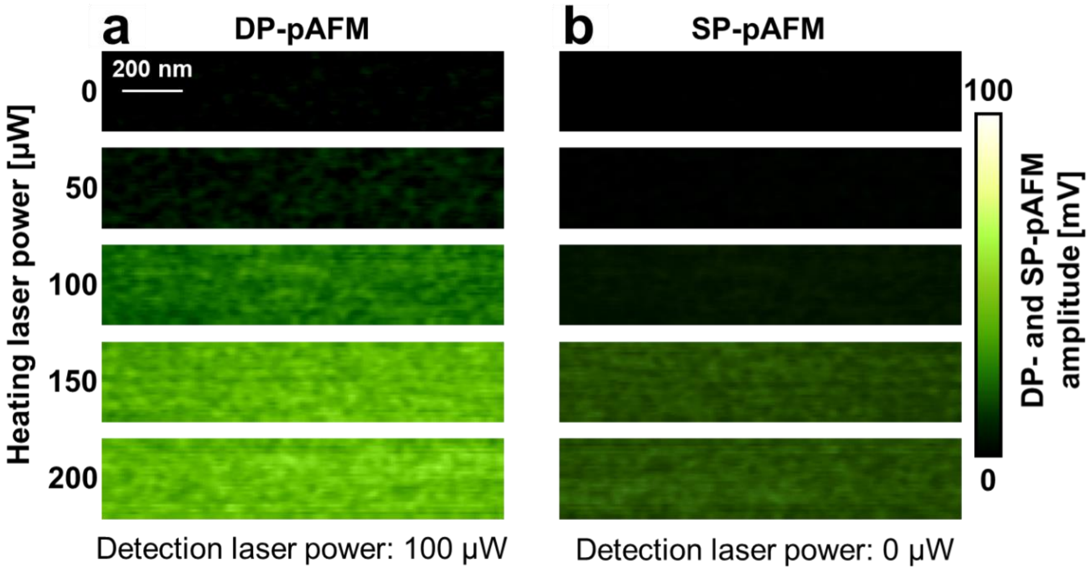

Supplementary Figure 1. DP- and SP-pAFM images of a DPP-DTT polymer sample, with 160 x 32 data points. (a) DP- and (b) SP-pAFM amplitude images captured while increasing the heating laser power from 0 to 200 μW at fixed detection laser powers of 100 and 0 μW, respectively. The total number of scanned points is 160 × 23. DP-pAFM, dual-pulse pAFM; SP-pAFM, single-pulse pAFM; DPP-DTT, diketopyrrolo-pyrrole-dithienylthieno [3,2-b]thiophene.

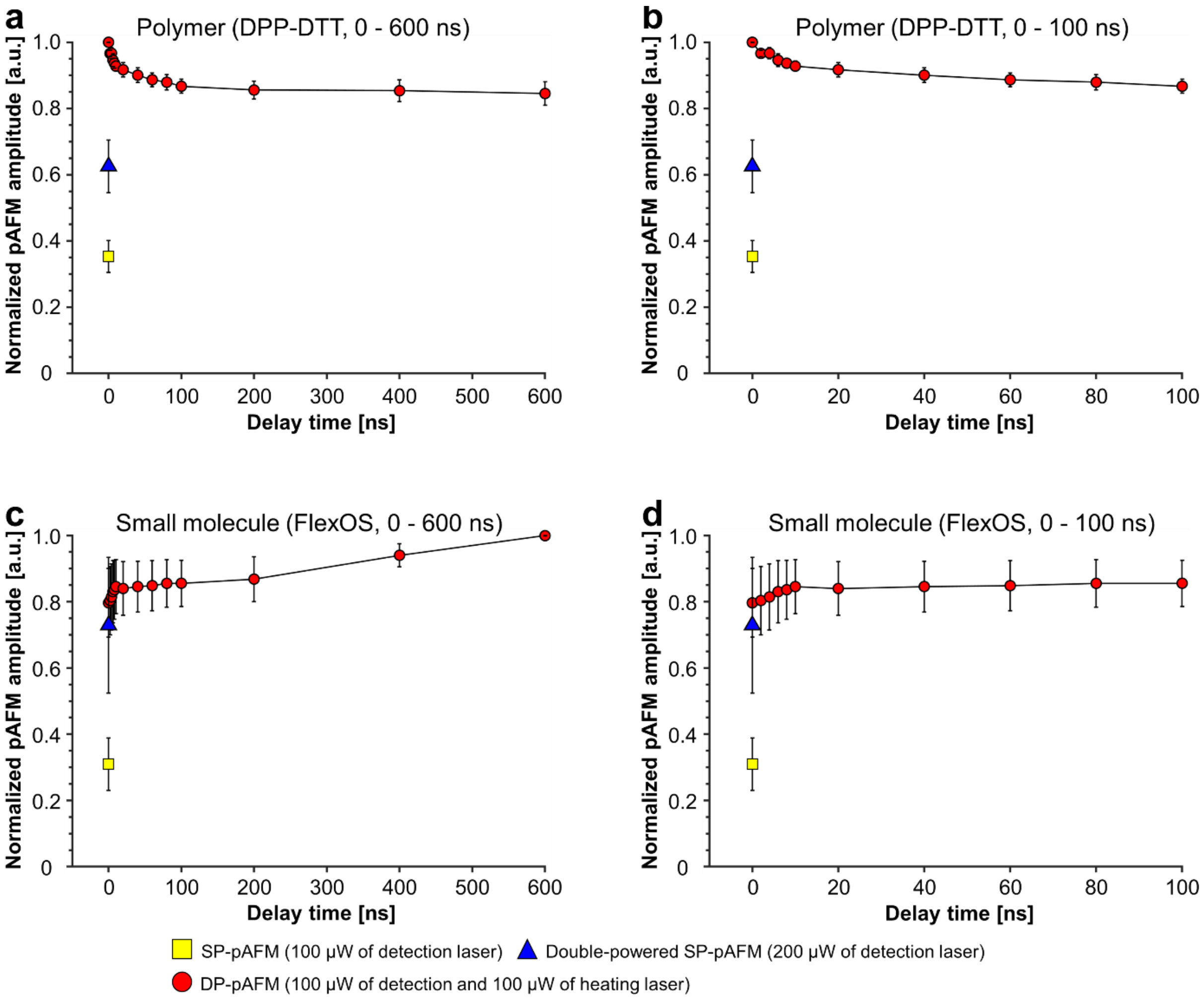

Supplementary Figure 2. Comparison of the signal amplitudes of SP-pAFM, double-powered SP-pAFM, and DP-pAFM in (a, b) DPP-DTT polymer and (c, d) FlexOS small molecule samples with time delays of less than 600 ns and 100 ns, respectively. SP-pAFM, single-pulse pAFM; DP-pAFM, dual-pulse pAFM.

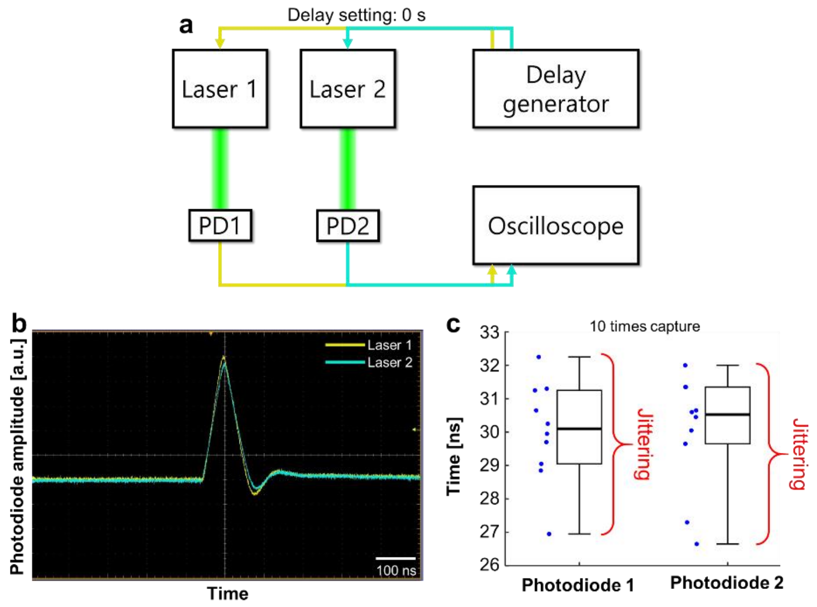

Supplementary Figure 3. Jittering measurement of heating and detection lasers. (a) Experiment schematic. (b) Photodiode responses of the two lasers captured by an oscilloscope. (b) Peak signals from two photodiodes

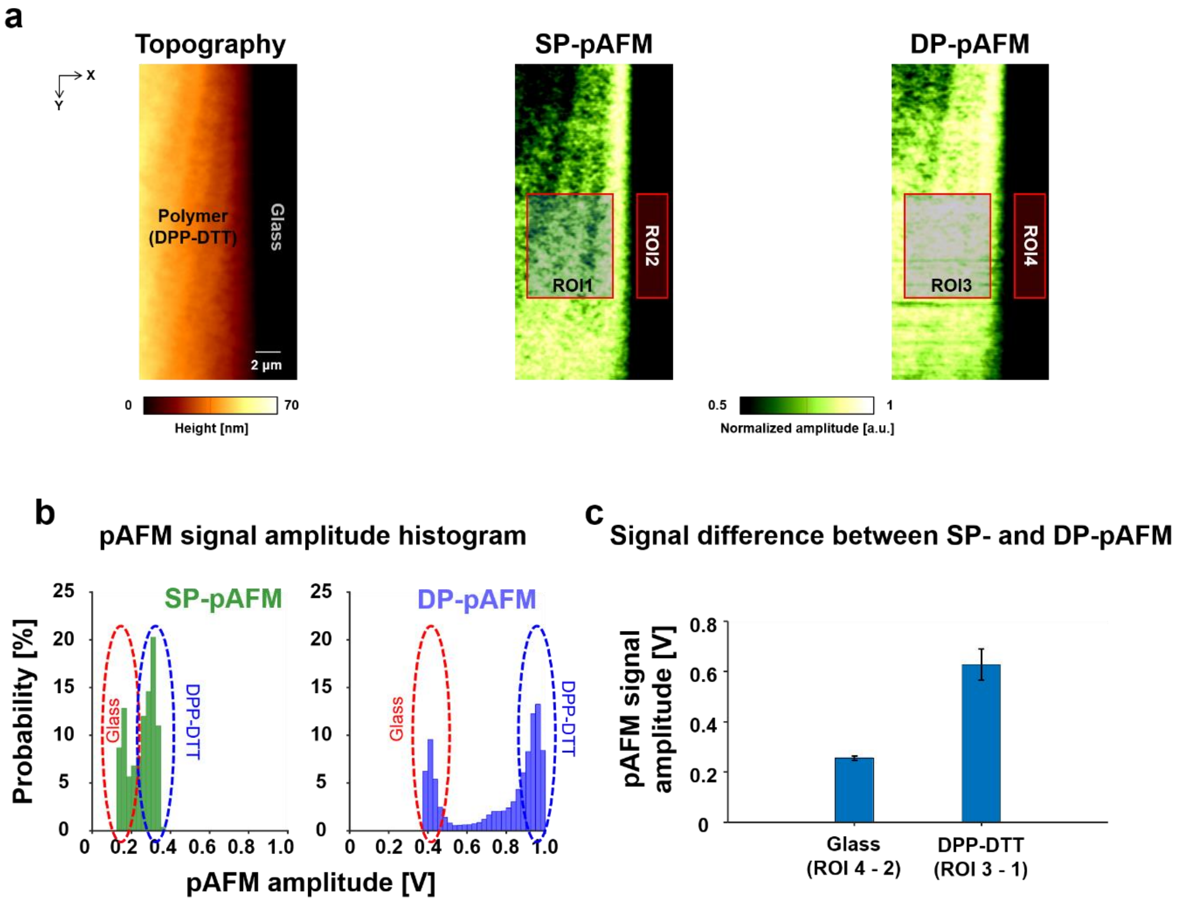

Supplementary Figure 4. Comparison of SP- and DP-pAFM signal amplitudes. (a) from left to right, AFM topography, SP-pAFM image, and DP-pAFM image of DPP-DTT polymer sample and glass substrate. (b) SP and DP-pAFM signal amplitude histograms. (c) Difference in signal amplitudes of SP and DP-pAFM. AFM, atomic force microscopy; SP-pAFM, single-pulse pAFM; DP-pAFM, dual-pulse pAFM.
