# Supplementary material for: Dual-pulse photoactivated atomic force microscopy
Source: Sci Rep. 2021 Aug 24;11:17097. doi: 10.1038/s41598-021-96646-4 (PMC8384876; doi:10.1038/s41598-021-96646-4)
Supplement: Supplementary file 3 — Supplementary Legend. [file 41598_2021_96646_MOESM3_ESM.docx]

**Supplementary movie 1.** The jittering of the heating and detection laser pulses.
